# Structural bioinformatics studies of bacterial outer membrane beta-barrel transporters and their AlphaFold2 predicted water-soluble QTY variants

**DOI:** 10.1371/journal.pone.0290360

**Published:** 2023-08-22

**Authors:** Akash Sajeev-Sheeja, Eva Smorodina, Shuguang Zhang

**Affiliations:** 1 Department of Chemistry, Indian Institute of Science Education and Research, Srinivasapuram, Yerpedu Mandal, Tirupati Dist, Tirupati, Andhra Pradesh, India; 2 Department of Immunology, Laboratory for Computational and Systems Immunology, University of Oslo, Oslo University Hospital, Oslo, Norway; 3 Laboratory of Molecular Architecture, Media Lab, Massachusetts Institute of Technology, Cambridge, MA, United States of America; Zhejiang University College of Life Sciences, CHINA

## Abstract

Beta-barrel outer membrane proteins (OMP) are integral components of Gram-negative bacteria, eukaryotic mitochondria, and chloroplasts. They play essential roles in various cellular processes including nutrient transport, membrane stability, host-pathogen interactions, antibiotic resistance and more. The advent of AlphaFold2 for accurate protein structure predictions transformed structural bioinformatic studies. We previously used a QTY code to convert hydrophobic alpha-helices to hydrophilic alpha-helices in over 50 membrane proteins with all alpha-helices. The QTY code systematically replaces hydrophobic leucine (L), isoleucine (I), valine (V), and phenylalanine (F) with hydrophilic glutamine (Q), threonine (T), and tyrosine (Y). We here present a structural bioinformatic analysis of five outer membrane beta-barrel proteins with known molecular structures, including **a**) BamA, **b**) Omp85 (also called Sam50), **c**) FecA, **d**) Tsx, and **e**) OmpC. We superposed the structures of five native beta-barrel outer membrane proteins and their AlphaFold2-predicted corresponding QTY variant structures. The superposed structures of OMPs and their QTY variants exhibit remarkable structural similarity, as evidenced by residue mean square distance (RMSD) values between 0.206Å to 0.414Å despite the replacement of at least 22% (Transmembrane variation) of the amino acids in the transmembrane regions. We also show that native outer membrane proteins and QTY variants have different hydrophobicity patches. Our study provides important insights into the differences between hydrophobic and hydrophilic beta-barrels and validates the QTY code for studying beta-barrel membrane proteins and perhaps other hydrophobic aggregated proteins. Our findings demonstrate that the QTY code can be used as a simple tool for designing hydrophobic proteins in various biological contexts.

## Introduction

Membrane proteins are essential for all cells, and they are vital for cells and living organisms to interact with their environments, both between internal cellular space and external surroundings. There are two general classes of membrane protein structural folds: **i**) predominately alpha-helices and **ii**) predominately beta-sheets, commonly referring as beta-barrels. Almost all eukaryote cellular membranes and bacterial inner membranes comprise alpha-helical transmembrane (TM) proteins. On the other hand, some bacterial outer membrane proteins (OMP) comprise only the integral beta-barrels [1,2]. Integral membrane proteins containing beta-barrel structures have been found in the outer membranes of gram-negative bacteria, mitochondria, and chloroplasts [3]. There are currently 129 distinct beta-barrel gene families with ~1.2 million entries for bacterial outer membrane proteins (http://www.ompdb.org) [4].

These OMPs have diverse functions, including pores for passive and active transport of molecules, ion channels [5], and antibiotic efflux channels that confer bacterial antibiotic resistance [6,7], bacterial cell defense, nutrient transport system, protein secretion, biogenesis, host cell adhesion, and invasion, biofilm formation (see a comprehensive review by [1]).

Beta-barrels are a class of protein structures that are commonly found in various bacterial outer membrane proteins [8]. Researchers have made progress in engineering protein nanopores using beta-barrels for DNA sequencing [9], small molecule detection [10], and targeted drug delivery for therapies [11]. Because OMPs are directly involved in antibiotic resistance, some recent research has focused their structure studies in order to discover new classes of antibiotics that specifically target the bacterial outer membrane proteins [6,7].

We carried out structural bioinformatic studies of five bacterial beta-barrel outer membrane proteins, they include **a**) BamA [12], **b**) Omp85 (= Sam50) [13,14], **c**) FecA [15], **d**) Tsx [16,17] and **e**) OmpC [18].

Bam refers to Barrel Assembly Machinery (BAM), and the central BamA subunit is an outer beta-barrel membrane protein in Gram-negative bacteria [12]. BamA promotes the membrane integration of partially folded beta-barrels by a ‘swing’ mechanism [19]. BamA is a member of the Omp85 superfamily, a group of 16-stranded β-barrel proteins implicated in membrane protein insertion and protein secretion processes in bacteria and organelles [19]. The β -barrel assembly machinery found in the outer membrane of Gram-negative bacteria is functionally equivalent to the Sorting and Assembly Machinery (SAM) complex, which comprises two critical proteins for cell survival: the channel-forming Sam50 and Sam35 [19,20]. Sam50 is responsible for folding and integrating β-barrel substrates into the outer membrane, and Sam35 interacts with the substrate β-signal located in the last β-strand [2]. Recent CryoEM structural analysis of the SAM complex in *Myceliophthora thermophila* reveals that Sam50 comprises a 16-stranded transmembrane β-barrel and a single polypeptide-transport-associated (POTRA) domain that extends into the intermembrane space [2]. Omp85 is an evolutionary conserved essential component of the protein insertion machinery. It is essential for outer membrane biogenesis in Gram-negative bacteria, eukaryotic mitochondria, and chloroplasts. Omp85 has some overlap function as BamA and was most likely required for the earliest stage of transforming endosymbiotic bacteria into eukaryotic organelles [13,14].

FecA is an iron(III) dicitrate transport outer membrane protein commonly found in *E*.*coli* and almost all bacteria. It serves as the outer membrane receptor protein in the Fe^3+^ dicitrate transport system [15]. In environments where ferric citrate is limited, FecA plays a crucial role in the survival of many bacteria by facilitating the uptake of nutrients [21]. FecA is also a regulatory protein that transduces a signal from the cell surface into the cytoplasm [22,23].

Tsx is an outer membrane transporter that exhibits nucleoside specificity found in Gram-negative bacteria, including *Escherichia coli* [16]. Tsx-nucleoside-specific channel-forming protein that facilitates the transport of nucleosides and deoxynucleosides across the outer membrane [24]. Tsx is a 10-stranded beta-barrel that creates a channel across the outer membrane. The presence of nucleosides controls the gating mechanism of the TSX protein, which opens and closes the channel to enable nucleoside transport [16,24].

OmpC is a trimeric structure in the bacterial outer membrane and belongs to the porin family of proteins. OmpC protein helps maintain the integrity of the outer membrane and protects the cell from harmful substances in the environment [25]. The beta-barrel structure of these proteins is critical to their function as it allows them to form a channel across the outer membrane and facilitate the transport of polyamines, including putrescine, cadaverine, spermidine, and spermine.

In this structural bioinformatics study, we used AlphaFold2, released in July 2021. Since its open access release, AlphaFold2 has been widely applied to accurately predict protein 3D structures and significantly accelerated protein structure studies. AlphaFold2 uses a deep neural network to predict the three-dimensional structure of proteins with remarkable accuracy, which is crucial not only for understanding their function but also for developing new molecular medicine [26]. In July 2022, DeepMind publicly released a database of >214 million protein structures, containing almost all known protein structures to date.

We previously applied the QTY (Glutamine, Threonine, Tyrosine) code to design several detergent-free alpha-helix transmembrane (TM) protein chemokine receptors and cytokine receptors for various uses [27–29]. The expressed proteins exhibited predicted characteristics, stable structures and retained their respective ligand-binding activity [27–29]. Later we carried out QTY variant protein structure predictions using AlphaFold2, achieving results in hours, instead of ~5 weeks for each molecular simulation using GOMoDo, AMBER and YASARA programs [27,28]. Furthermore, we also used AlphaFold2 to predict water-soluble QTY variants of the 14 glucose transporters [30] and 13 solute carrier transporters [31]. We directly compared these QTY variants with their native protein structures.

We recently asked if the QTY code is also applicable to beta-sheet structures. Since beta-barrel outer membrane proteins comprise mostly bets-sheets, we ask if QTY code can also be used to design water-soluble beta-barrel outer membrane protein variants.

Here we report structural bioinformatics studies of the molecular structures of five experimentally-determined beta-barrel outer membrane proteins and their AlphaFold2-predicted water-soluble QTY variants. The native beta-barrel OMP structures and their QTY variants share remarkable structural similarities and superpose very well with residue mean-square distances (RMSD) between 0.206Å to 0.414Å despite the replacement of at least 22% transmembrane amino acids. We also show that native outer membrane proteins and QTY variants have different hydrophobicity patches, and the QTY variants have more hydrophilic surfaces. Our study not only provides important insights into the differences between hydrophobic and designed hydrophilic beta-barrels, but also it validates the QTY code for studying beta-barrel membrane proteins and perhaps other hydrophobic aggregated proteins. Our findings demonstrate the QTY code as a simple tool not only for studying alpha-helix membrane proteins but also mostly beta-sheet proteins.

## Results and discussions

### Protein sequence alignments and other characteristics

**Fig 2:**
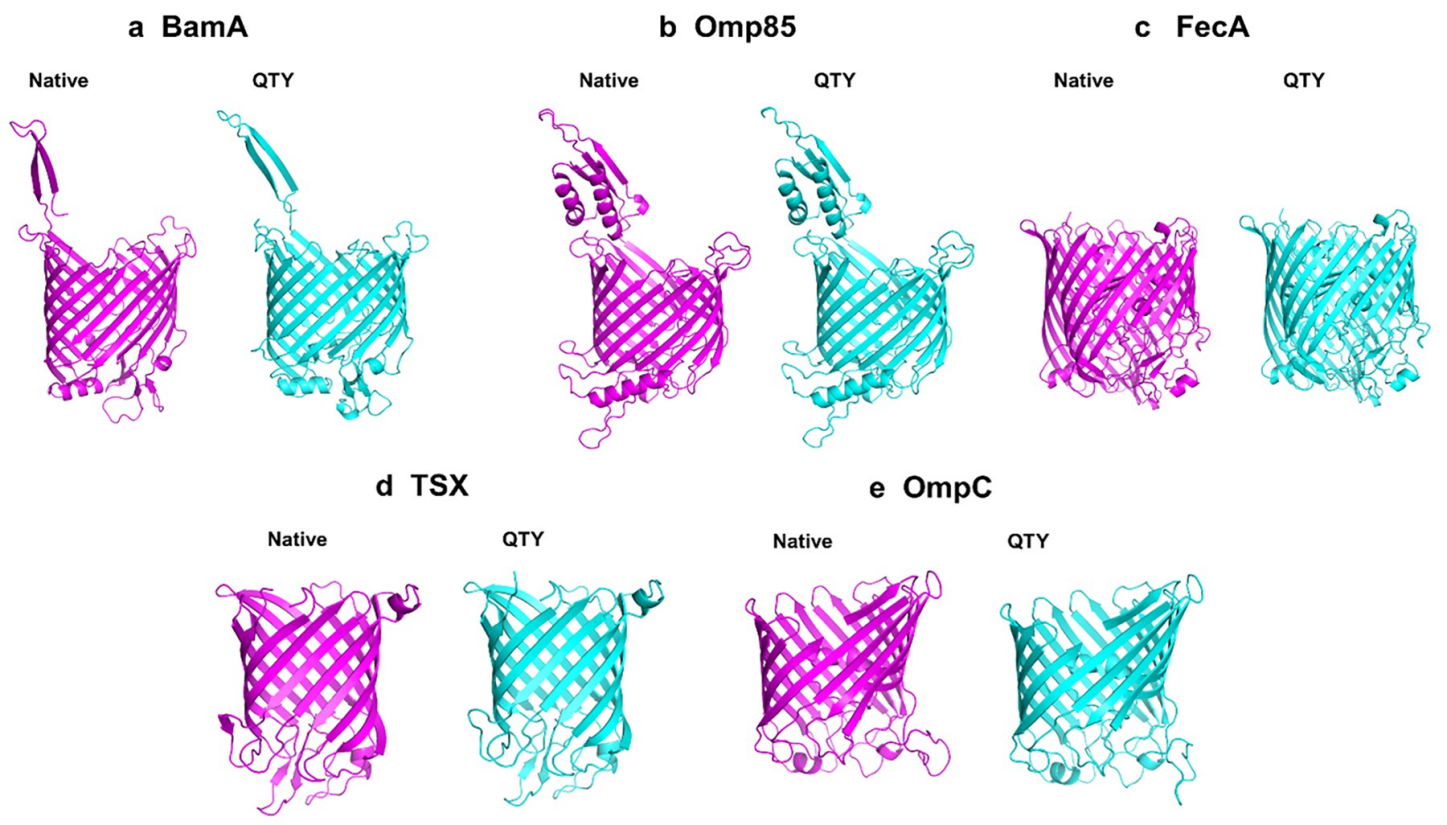
Structures of the five bacterial native outer membrane beta-barrel proteins and their AlphaFold2-predicted water-soluble QTY variants. The crystal and CryoEM native integral outer membrane beta-barrel structures are coloured magenta, the water-soluble QTY variant structures are colored cyan. These proteins are shown in the identical orientations. **a**, BamA *vs* BamA^QTY^, **b**, Omp85 (= Sam50) *vs* Omp85^QTY^, **c**, FecA *vs* FecA^QTY^, **d**, Tsx *vs* Tsx^QTY^, and **e**, OmpC *vs* OmpC^QTY^.

**Fig 3:**
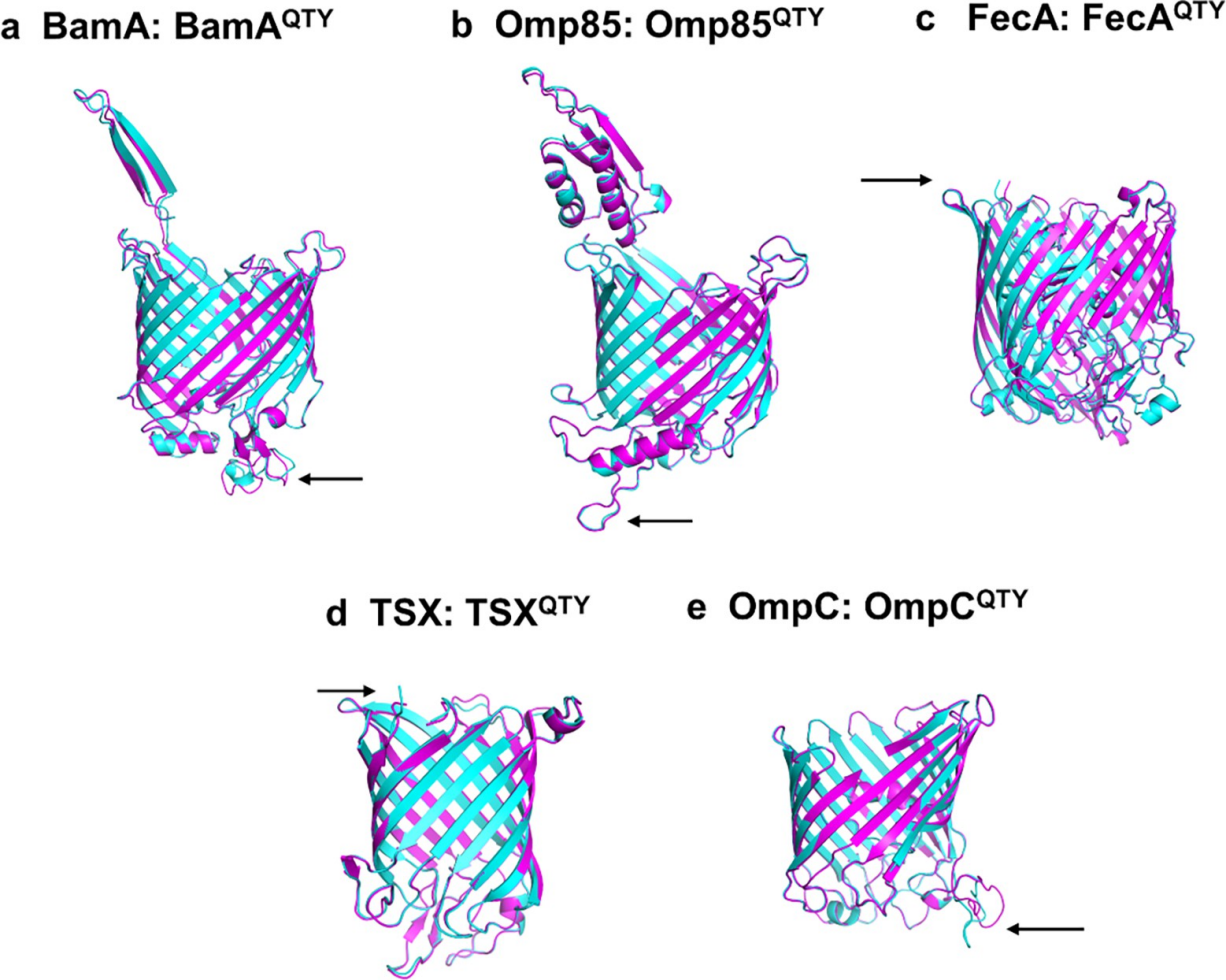
Superposed five bacterial native outer membrane beta barrel proteins and their AlphaFold2-predicted water-soluble QTY variants. The native structures (magenta) and their water-soluble QTY variants (cyan) are shown with arrows pointing to the deviations in unstructured loops. For the superposed structures, the RMSD is in Å (). These superposed structures are shown: **a**, BamA *vs* BamA^QTY^ (0.414Å), **b**, Omp85 (= Sam50) *vs* Omp85^QTY^ (0.322Å), **c**, FecA *vs* FecA^QTY^ (0.256Å), **d**, Tsx *vs* Tsx^QTY^ (0.307Å), **e**, OmpC *vs* OmpC^QTY^ (0.206Å).

The QTY code was conceived to replace four hydrophobic amino acids (leucine (L), isoleucine (I), valine (V), and phenylalanine (F) with three neutrally polar amino acids (glutamine (Q), threonine (T), and tyrosine (Y). The transmembrane segments’ hydrophobic amino acids L, I/V, and F are systematically pairwise replaced with Q, T, and Y residues. The overall variations observed in the proteins after applying the QTY code to the native proteins are presented in Table 1. Interestingly, these variations had minimal impact on the protein structure (Figs 2 and 3).

**Table 1:** Characteristics of bacterial native outer membrane beta-barrel proteins and their water-soluble QTY variants.

Name	RMSD	pI	MW(kDa)	^§^TM variations(%)	^¶^Overall variations(%)
BamA Q5F5W8	___	9.19	43.36	___	___
BamA^QTY^	0.414Å	9.10	43.81	27.22 (49/180)	12.25
Omp85 (= Sam50) G2QFF9	___	9.35	48.78	___	___
Omp85^QTY^	0.322Å	9.22	49.25	32 (45/141)	9.65
FecA P13036	___	5.47	73.36	___	___
FecA^QTY^	0.256Å	5.47	73.93	22.6 (71/314)	10.74
Tsx P0A927	___	5.23	30.87	___	___
Tsx^QTY^	0.307Å	5.23	31.11	25.69 (37/144)	13.85
OmpC P06996	___	4.48	38.30	___	___
OmpC^QTY^	0.206Å	4.48	38.61	23.75 (43/181)	12.42

Note: ^§^TM variations refers to the percentage of changed residues within the transmembrane (TM) domains of the respective proteins. The values in parentheses represent the percentage of TM variations, calculated as the number of changed residues in the transmembrane domains over the total number of residues within the TM domains.

^¶^Overall variations- represents the number of changed amino acids replaced with the QTY code over the total amino acid count in the protein.

**Fig 1:**
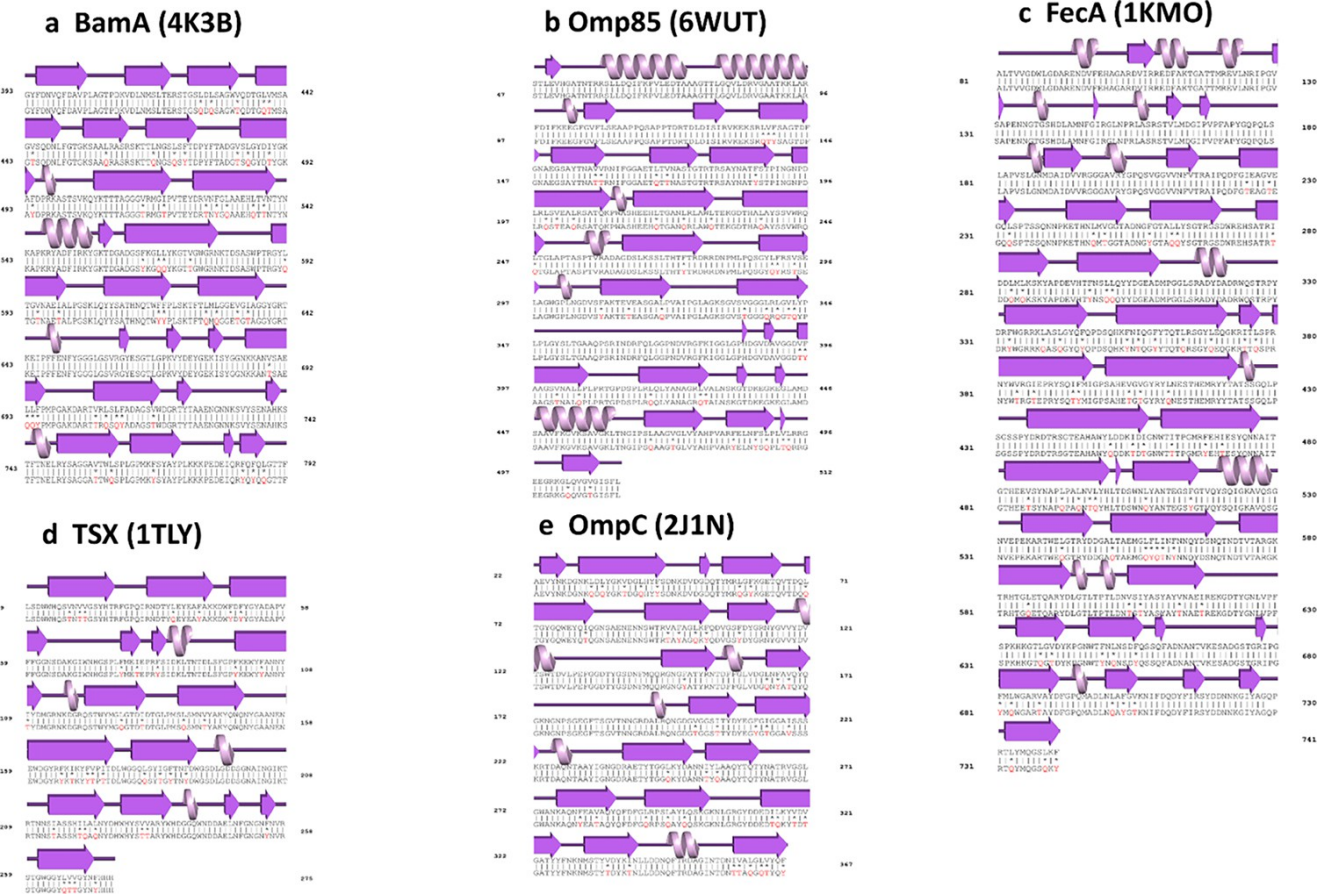
Protein alignments of five bacterial outer membrane beta-barrel protein sequences and their water-soluble QTY variants. The symbols | and * indicate the identical and different amino acids, respectively. Please note the Q, T and Y amino acid replacement (red). The Beta-sheets (magenta) are shown above the protein sequences. A few alpha-helices also exist in the membrane beta-barrel proteins. But they are not subjected to the QTY code designs. The alignments are: **a**, BamA *vs* BamA^QTY^, **b**, Omp85 *vs* Omp85^QTY^, **c**, FecA *vs* FecA^QTY^, **d**, Tsx *vs* Tsx^QTY^, and **e**, OmpC *vs* OmpC^QTY^. For additional information, please see S1 Fig in S1 File.

We aligned native outer membrane transporters that have crystal and Cryo-EM structures with their QTY variants (Fig 1 and S1 Fig in S1 File). Despite major QTY replacement of hydrophobic residues in the transmembrane domains (22%-32%) in the outer membrane beta-barrel transporters, the isoelectric focusing point pI and molecular weight remain similar (Table 1). This is because the Q, T, and Y amino acids are neutral without any positive and negative charges, but they have polar properties that make them hydrophilic in nature, so they introduce water-soluble side chains. The sidechains -OH of T (threonine) and Y (tyrosine) can form 3 hydrogen bonds with water molecules, 1 donor from H (hydrogen), and 2 acceptors from O (oxygen). The side chains -NH_2_ of Q (glutamine) form 4 hydrogen bonds with water, 2 donors from -NH_2,_ and 2 acceptors through oxygen on -C = O. Therefore, the hydrophobicity of the transmembrane beta-barrels is noticeably reduced. For example, transmembrane beta-sheets in protein sequences of Omp85 and FecA exhibit differences of 32% and over 22% compared to their water-soluble QTY variants, respectively (Table 1).

The selected targets span a range of isoelectric-focusing points (pIs), with some falling in the acidic range while others fall within a similar range. It is noted that the pIs are identical for several native and QTY variants. Two have basic pI, namely, BamA (pI 9.19), and Omp85 (pI 9.35). On the other hand, three have mild acidic pI, for example, FecA (pI 5.47), TSX (pI 5.23) and OmpC (pI 4.48), despite the large number of QTY amino acid substitutions. The amino acids glutamine, threonine, and tyrosine (Q, T, Y) have neither positive nor negative charges at neutral pH. The isoelectric point (pI) of the protein is almost unaffected by substitutions involving these residues. This observation is particularly significant since changes in pI can cause non-specific protein interactions, underscoring the need to carefully consider the impact of specific amino acid substitutions on protein function and behaviour.

The method of X-ray crystallography is commonly used to determine the three-dimensional structure of proteins at atomic resolution [32]. This technique relies on the measurement of electron density maps derived from X-ray diffraction patterns. By analysing these electron density maps, it becomes possible to define and arrange amino acids within the protein structure. The electron density map is compared to a library of known amino acid structures to determine which amino acids are present in the protein structure. This comparison involves fitting the amino acid models into the electron density map and assessing the compatibility between the experimental data and the proposed amino acid placement [33]. As shown in the electron density maps [34], Leucine (L) with glutamine (Q), isoleucine (I) and valine (V) with threonine (T), and phenylalanine (F) with tyrosine (Y) are among the pairs of amino acids with the greatest structural similarity. Readers can visit Wikipedia to refer more about the QTY code. By replacing the CH3- on Lue and Val with -OH groups on Gln (Q) and Thr (T), as well as by adding an OH- group to phenylalanine (F) to become Tyr (Y), the transmembrane beta-sheets have undergone QTY substitutions that range from 22–32%. The QTY substitutions in the transmembrane beta sheets showed minor structural changes based on visual inspection (Fig 2) and RMSD values (Table 1). These substitutions slightly increased the molecular weight of each of the proteins (Table 1). These superposed beta-barrel structures look to be very similar (Fig 3).

### Superposition of native beta-barrel transporters and their water-soluble QTY variants

We superposed the native outer membrane beta-barrel transporters determined by X-ray crystallographic or CryoEM with their corresponding QTY variants (Fig 3). The molecular structures of native beta-barrel membrane transporters are available for BamA (PDB: 4K3B), Omp85 (PDB: 6WUT), FecA (PDB: 1KMO), TSX (PDB: 1TLY) and OmpC (PDB: 2J1N). We superposed the experimentally determined molecular and QTY structures (Fig 3). The beta-barrel transmembrane proteins of the experimentally determined native structures (magenta colour) and the AlphaFold2 predicted water-soluble QTY variants (cyan colour) exhibit a significant degree of similarity, as shown in Fig 3. These superposed beta-barrel structure results suggest that the QTY code is applicable for beta-sheet structures. However, as expected, there are some deviations in unstructured loops since AlphaFold2 is less capable of predicting the unstructured loops (Fig 3), the arrows are used to indicate deviations in unstructured loops.

### Analysis of the hydrophobic surface of native beta barrel transporters and the water-soluble QTY variants

The hydrophobicity levels of native beta-barrel transmembrane segments are well established. The solubility and stability of beta-barrels vary depending on the protein and its surroundings. Because these integral beta-barrel transmembrane proteins are intrinsically hydrophobic, they require surfactants for solubilization and stabilization in water after removing them out of the integral membrane. In the native crystal beta-barrels, beta-sheets are directly embedded in the hydrophobic lipid bilayer, with hydrophobic side chains of isoleucine, leucine, valine, and phenylalanine interfacing with the lipid bilayer.

**Fig 4:**
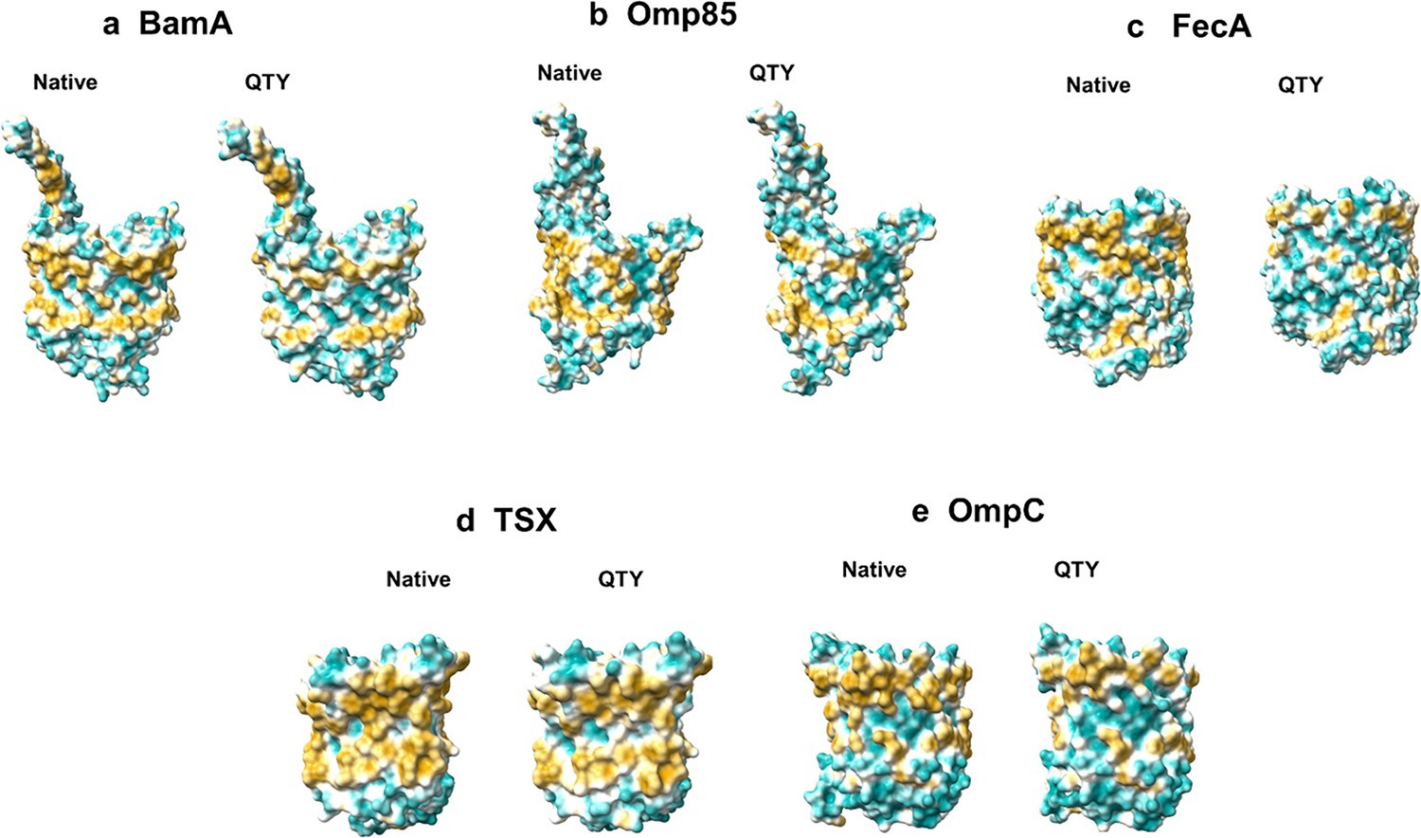
Hydrophobic surfaces of five bacterial outer membrane beta barrel proteins and their AlphaFold2 predicted water-soluble QTY variants. After Q, T, and Y replacement of the hydrophobic residues L, I, V, F, the protein surfaces become more hydrophilic. The hydrophobic surface (brownish) of the native beta-barrel transporters became more cyan colour indicating the hydrophobic surface is largely reduced on the transmembrane helices for the QTY variants. These proteins are set side by side for direct comparison: **a**, BamA *vs* BamA^QTY^, **b,** Omp85 (= Sam50) *vs* Omp85^QTY^, **c,** FecA *vs* FecA^QTY^, **d,** Tsx *vs* Tsx^QTY^, and **e**, OmpC *vs* OmpC^QTY^. For further information on the changes in hydrophobicity, please see S2 Fig in S1 File.

The hydrophobic surfaces are lowered by substituting hydrophobic amino acids such as L, I/V, and F with hydrophilic amino acids Q, T, and Y [27,34]. The changes in hydrophobicity resulting from the QTY conversion from hydrophobic to hydrophilic beta-sheets are shown in Fig 4 and S2 Fig in S1 File.

The basis for the QTY code’s pairwise replacement of protein secondary structures from amino acid sequences is a shared molecular structure prevalent in several protein types. Based on their chemical features, beta-sheets can also be classified into three chemically distinct types. Type I beta-sheets are water-soluble, hydrophilic structures found in globular proteins such as green fluorescent protein (GFP) [35]. In contrast, Type II beta-sheets are water-insoluble, hydrophobic structures found in beta-barrel membrane proteins [1,2]. Amphiphilic type III beta-sheets, which contain both hydrophobic and hydrophilic faces, are found in silk proteins [36,37]. Regardless of their chemical properties, all varieties of beta-sheets have a similar structure, with strands joined by hydrogen bonds on the backbones [38].

### AlphaFold2 predictions

Accurately predicting protein folding has been a holy grail in structural biology and protein science since 1960s, and numerous efforts have been made. However, this task has been extremely challenging until the emergence of AlphaFold2, which is an AI and machine learning-based tool. AlphaFold2 can accurately predict protein 3D structures. This tool has made it possible to investigate previously inaccessible protein structures, particularly those embedded in cell membranes, and for protein design, leading to significant advancements in studying proteins.

Our current study using AlphaFold2 has shown that the water-soluble QTY-variant structures of bacterial outer membrane beta-barrel transporters closely resemble their native structures. Our results further validate that the QTY code is likely applicable to other transmembrane proteins, including mostly alpha-helix and mostly beta-sheets.

## Conclusion

Furthermore, World Health Organization (WHO) warned that the recent antibiotic resistance bacteria worldwide are on the rise [39], and these bacteria have caused numerous unnecessary extended hospital-stay and unfortunate patient death [40]. A few new classes of antibiotics are urgently needed [41]. Thus, it is possible to use the water-soluble QTY variant beta-barrel proteins as targets and research reagents for the discovery of a new class of antibiotics that can block these essential bacterial outer membrane beta-barrels to combat bacterial antibiotic resistance.

The native structures of the proteins analysed in this study remained unaffected by commonly employed purification techniques. Solubilization in detergent, which can displace crucial lipids necessary for stability, as well as the use of purification tags, known to interfere with oligomeric assembly, did not impact these native structures. Purification tags can interfere with the formation of salt bridges between the N- and C-termini of porins [42]. To overcome this limitation, *Aunkham et al* purified the untagged form of Vibrio harveyi chitoporin to successfully determine the trimeric structure of the protein. Among the five targets selected in this study, two were based on proteins expressed with purification tags: BamA (4K3B) featuring an N-terminal His10 tag and Tsx (1TLY) featuring a C-terminal His6-tag. In contrast, Omp85, FecA, and OmpC were purified without any tags. All five proteins had been detergent-solubilized, which removes the annular lipids. It is important to consider these aspects, as they may have implications for antibiotic discovery. However, the future availability of structures for non-tagged proteins, along with their associated annular lipids, as achieved through extraction with native nanodisc polymers [43]. Conducting experimental validation of the QTY predictions for beta-barrel proteins would offer valuable insights into their potential to generate soluble and active proteins.

## Methods

### Protein sequence alignments and other characteristics

The native protein sequences for beta-barrel outer membrane transporters and their QTY-variant sequences were aligned using the same methods previously described. We used the Expasy website (https://web.expasy.org/compute_pi/) to perform calculations of the proteins’ molecular weights (MW) and isoelectric points (pI).

### AlphaFold2 prediction

AlphaFold2 Program https://github.com/sokrypton/ColabFold was used for the structure predictions of the QTY variants following the instructions at the website on 11th Gen Intel Core i5-11300H Quad-Core Processor, 16GB GB RAM, and Iris Xe Graphics, 512GB NVMe SSD. All the structures predicted by AlphaFold2 are available on the European Bioinformatics Institute (EBI) website (https://alphafold.ebi.ac.uk). The protein identification, entry name, description, and FASTA sequence for each protein can be found on the Uniprot website (https://www.uniprot.org). To obtain the data, a custom Python code was used to extract information from UniProt.

### Superposed structures

The molecular structures are from PDB https://www.rcsb.org. They include BamA (PDB:4K3B), Omp85 (PDB: 6WUT), FecA (PDB: 1KMO), Tsx (PDB: 1TLY), and OmpC (PDB: 2J1N). AlphaFold2 predictions of outer membrane beta-barrel transporters QTY variants were carried out using the AlphaFold2 at https://github.com/sokrypton/ColabFold. Uniprot https://www.uniprot.org is the source for the native beta-barrel outer membrane transporter protein sequences, and AlphaFold2 was performed to predict QTY variant structures. These structures are superposed using PyMOL https://pymol.org/2/.

### Structure visualization

For visualizing the structures, two primary software tools were employed: PyMOL (https://pymol.org/2/) and UCSF ChimeraX 1.4 (https://www.rbvi.ucsf.edu/chimera/). PyMOL was utilized for superimposing the models, whereas ChimeraX 1.4 was used to generate hydrophobicity models.

## Supporting information

S1 File Contains all the supporting figures. (DOCX)
