# Supplementary material for: Structural bioinformatics studies of bacterial outer membrane beta-barrel transporters and their AlphaFold2 predicted water-soluble QTY variants
Source: PLoS One. 2023 Aug 22;18(8):e0290360. doi: 10.1371/journal.pone.0290360 (PMC10443868; doi:10.1371/journal.pone.0290360)
Supplement: S1 File — (DOCX) [file pone.0290360.s001.docx]

**Supporting Information**

**Figure S1. Enlarged panels for Figure 1**. Enlarged protein sequences alignments of the outer membrane beta-barrel and their QTY variants. The readers can see the five native protein and QTY code variant sequences and understand how the QTY code works.

a) BamA, [Uniprot](https://www.uniprot.org/): [Q5F5W8](https://www.uniprot.org/uniprotkb/Q5F5W8/entry), **(**PDB: [4K3B](https://www.rcsb.org/structure/4K3B)**)**


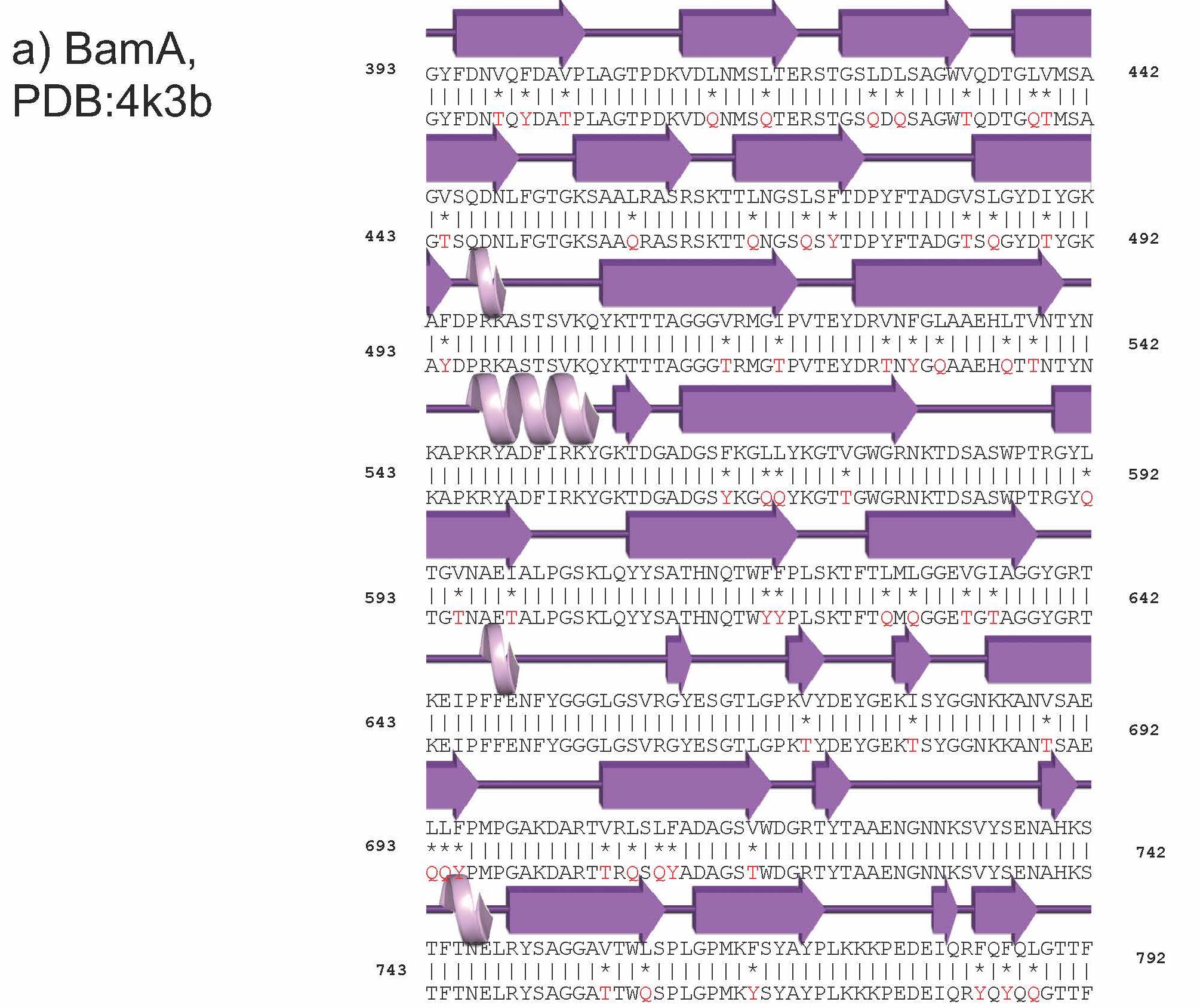


**BamA**

**PDB: 4K3B**

**b)** Omp85, [Uniprot](https://www.uniprot.org/): [G2QFF9](https://www.uniprot.org/uniprotkb/G2QFF9/entry) (PDB: [6WUT](https://www.rcsb.org/structure/6WUT))

**OMP85**

**PDB: 6WUT**

**
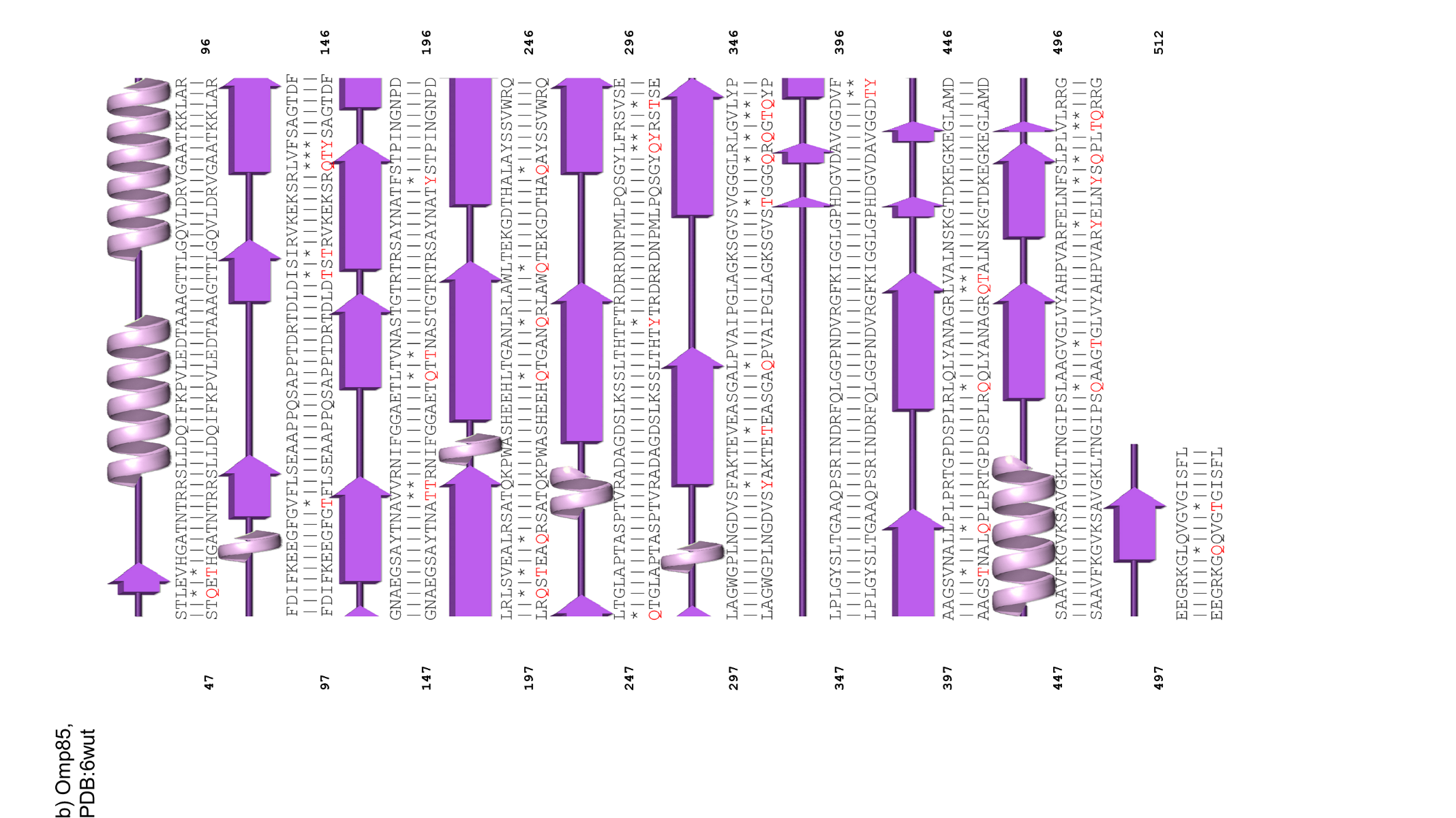
**

**c)** FecA, [Uniprot](https://www.uniprot.org/): [P13036](https://www.uniprot.org/uniprotkb/P13036/entry) (PDB: [1KMO](https://www.rcsb.org/structure/1KMO)),

**FecA**

**PDB: 1KMO**

**
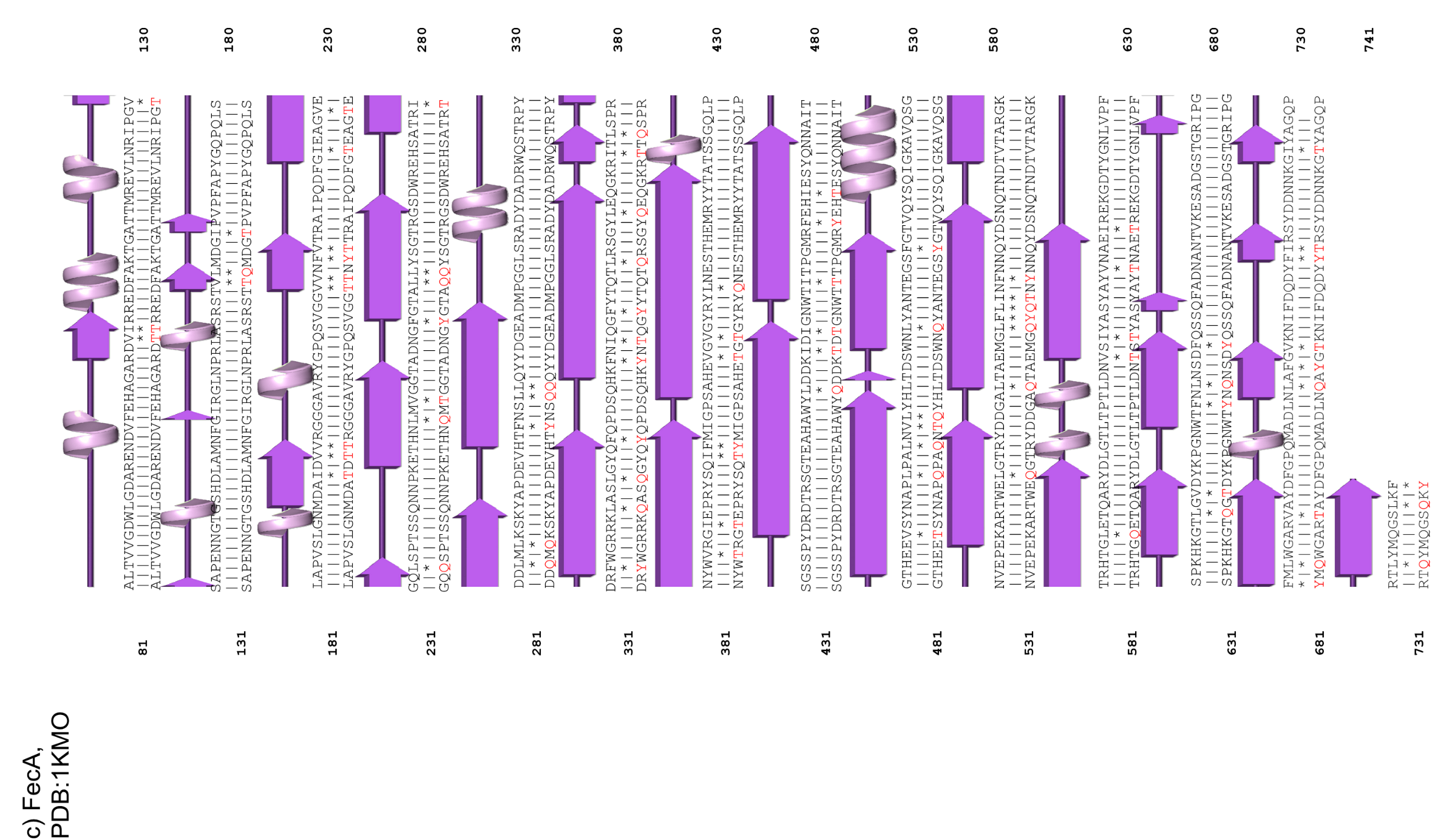
**

**d)** Tsx, [Uniprot](https://www.uniprot.org/): [P0A927](https://www.uniprot.org/uniprotkb/P0A927/entry) (PDB: [1TLY](https://www.rcsb.org/structure/1TLY))

**
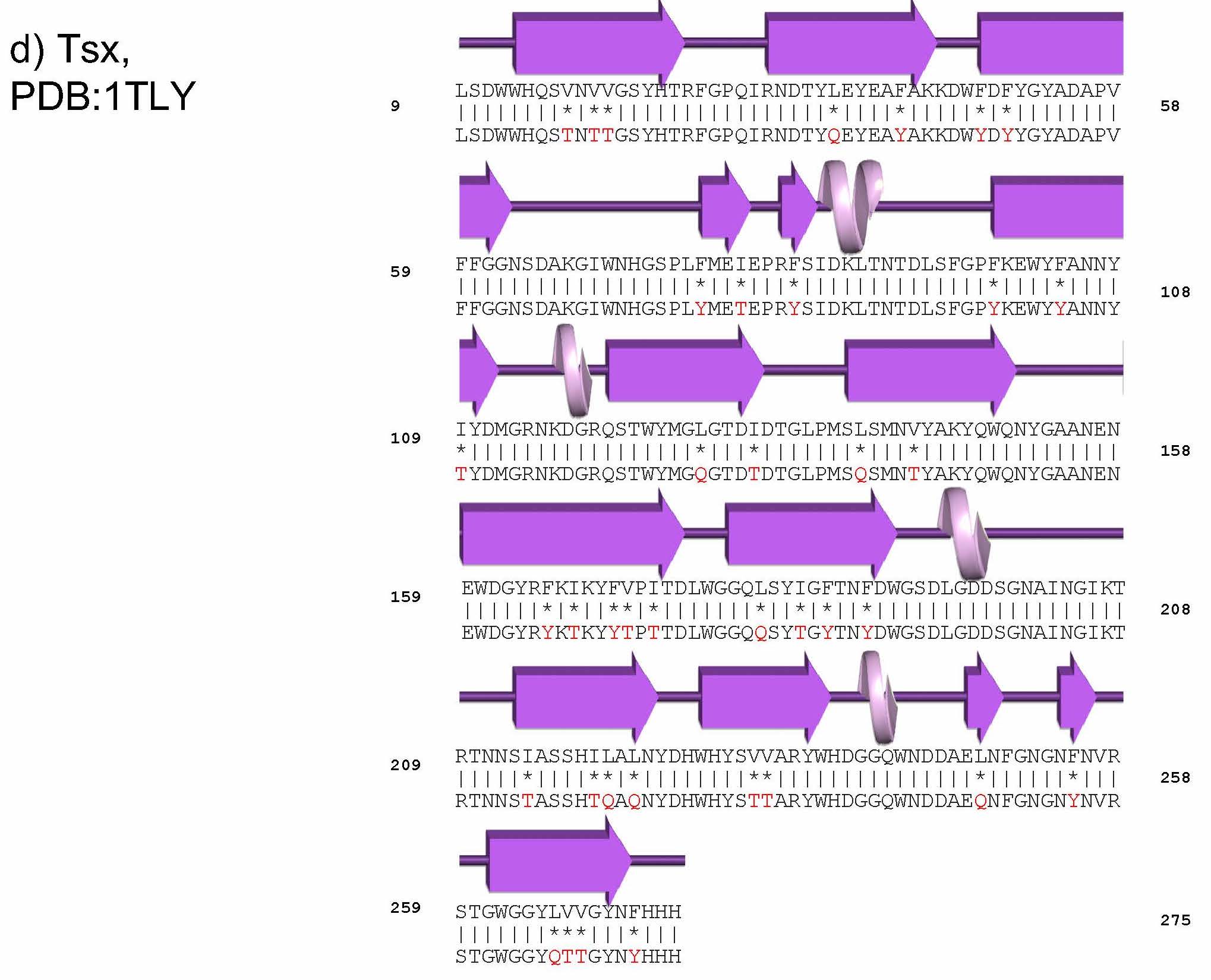
**

**TSX**

**PDB: 1TLY**

**e)** OmpC, [Uniprot](https://www.uniprot.org/): [P06996](https://www.uniprot.org/uniprotkb/P06996/entry) (PDB: [2J1N](https://www.rcsb.org/structure/2J1N))

**
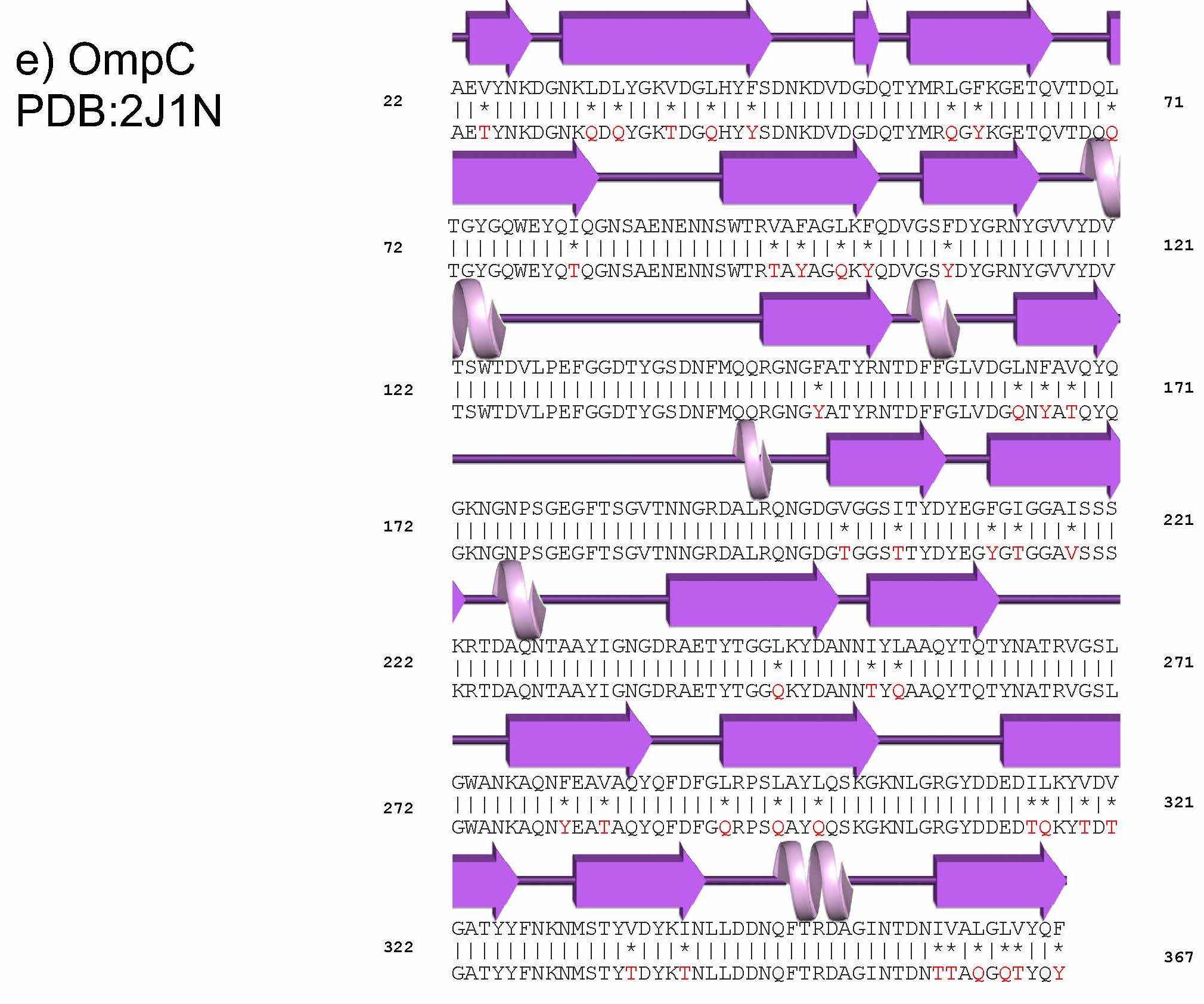
**

**OmpC**

**PDB: 2J1N**

**Figure S2. Hydropathy plots of Outer Membrane Beta-Barrel Native *vs.* QTY Variants to confirm the changes in hydrophobicity**. A clear distinction in hydrophobicity becomes evident when comparing hydropathy plots of the native proteins with their corresponding QTY variants of the five OMPs**.** The x-axis shows the position of amino acid residues in the protein sequence, while the y-axis (score) represents the hydrophobicity or hydrophilicity of those residues. Higher values along the y-axis indicate greater hydrophobicity, while lower values indicate increased hydrophilicity—window size: adjacent amino acid residues used for hydrophobicity calculation in hydropathy plots.

[Expasy - ProtScale](https://web.expasy.org/protscale/) **window size=9**

**a) BamA (PDB: 4K3B) 395-795 part (400 Amino Acids**)


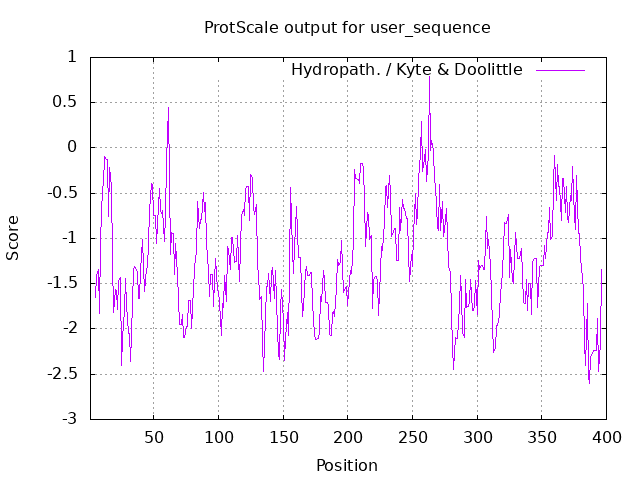

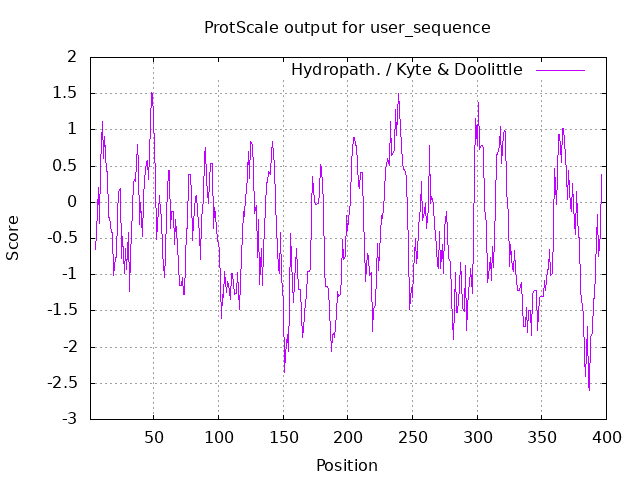
**Native** **QTY**

**b) Omp85 (PDB: 6WUT) 47 to 512 (466 Amino Acids)**

**Native** **QTY**


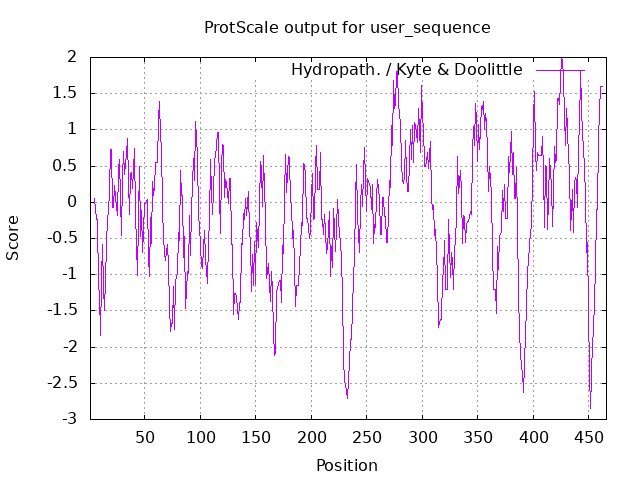

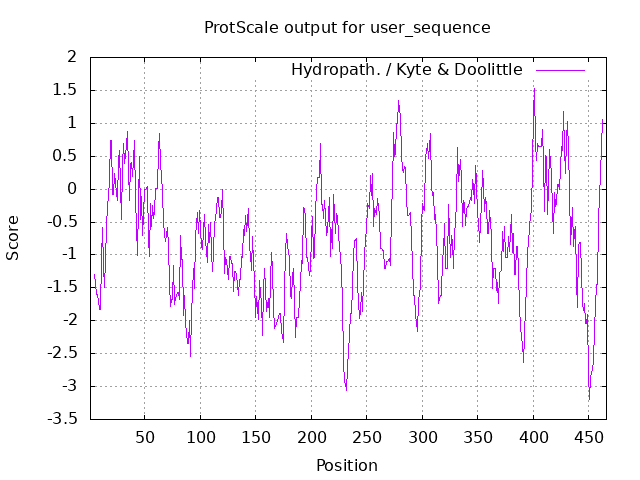


**c) FecA (PDB: 1KMO)** **81-741 part (661 Amino Acids)**


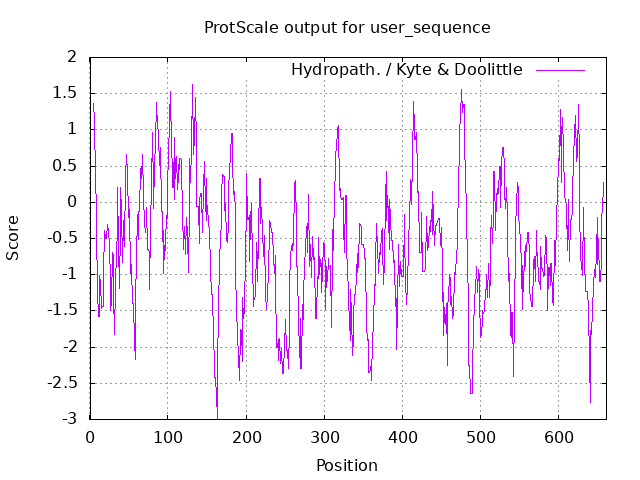
**Native** **QTY**


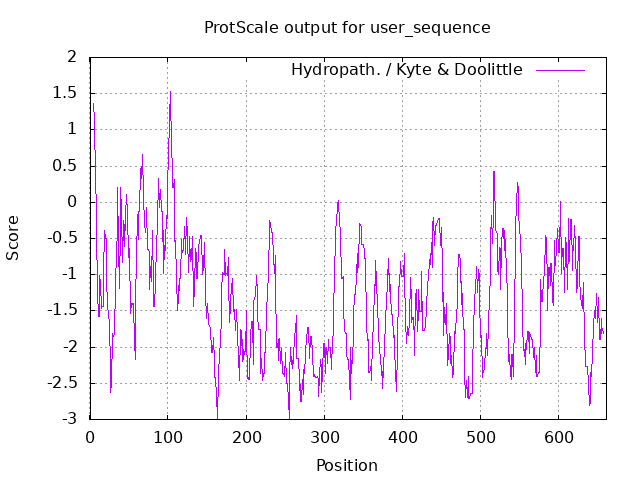


**d)TSX (PDB: 1TLY) 9-275aa (267 Amino acids)**


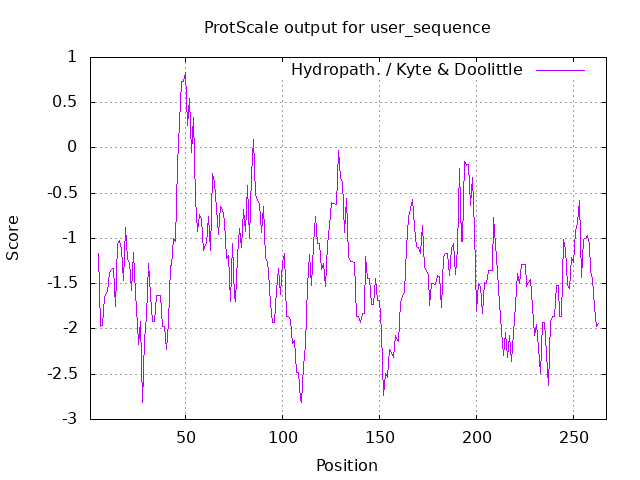

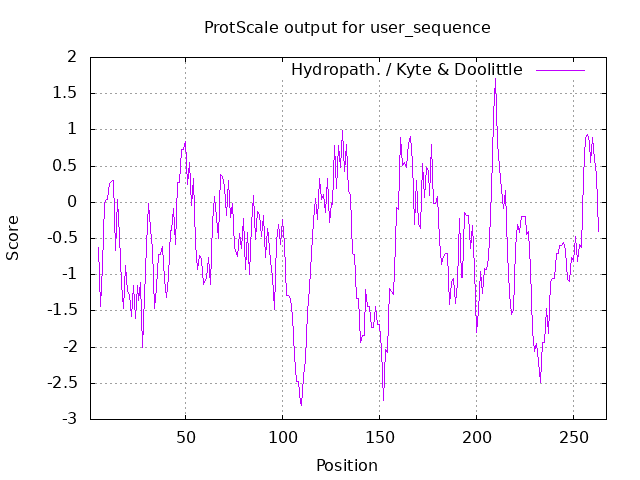
**Native** **QTY**

**e)OmpC (PDB: 2J1N) 22-367- part (346Amino Acids)**


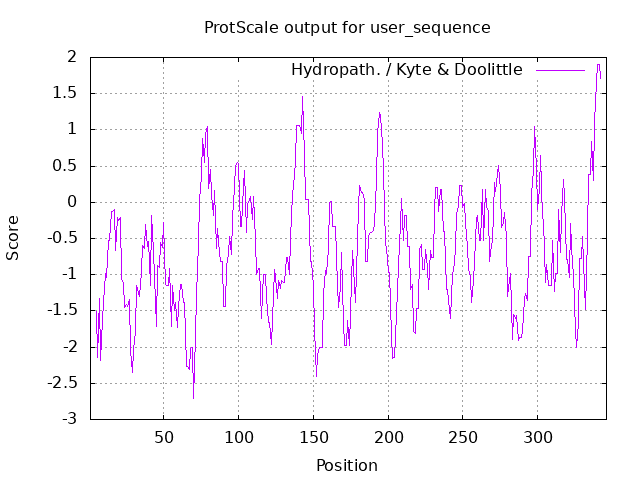

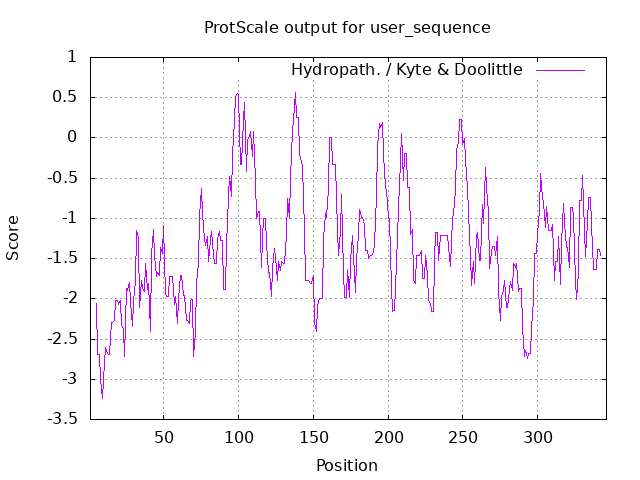
**Native** **QTY**
